# Temperature-Dependent Atomic Layer Deposition of Passivating ZnO Nanolayers for Dye-Sensitized Solar Cells

**DOI:** 10.3390/nano15241891

**Published:** 2025-12-17

**Authors:** Elizabeth Adzo Addae, Marek Szindler, Wojciech Sitek, Krzysztof Matus

**Affiliations:** 1 Scientific and Didactic Laboratory of Nanotechnology and Material Technologies, Faculty of Mechanical Engineering, Silesian University of Technology, Towarowa 7 Str., 44-100 Gliwice, Poland; marek.szindler@polsl.pl (M.S.); wojciech.sitek@polsl.pl (W.S.); 2 Materials Research Laboratory, Silesian University of Technology, Konarskiego 18a Str., 44-100 Gliwice, Poland

**Keywords:** Zinc oxide (ZnO), atomic layer deposition (ALD), deposition temperature, microstructure, thickness

## Abstract

The influence of ZnO nanolayers as a passivating layer prevents electrons from recombining with the electrolyte or oxidized dye molecules at the interface by acting as a blocking layer for semiconducting materials. At 300 °C, it was observed that FTO-ZnO 500-cycle samples recorded the lowest Rq and Ra values of 1210 nm and 0.877 nm, respectively, resulting in homogeneous, crystalline, and smooth surface thin films. SEM images of FTO-ZnO 500 cycles-300 °C (150.00 KX) show a much more crystalline and homogeneous layer, while FTO-ZnO 500 cycles-100 °C (150.00 KX) show an irregular and agglomerated surface. Energy-dispersive spectroscopy also revealed that ALD successfully deposited ZnO on the FTO glass substrates, especially at 300 °C, resulting in uniform layers. In visible light wavelength (400 nm–800 nm), FTO-ZnO 500 cycles-300 °C exhibited the highest stable transmittance value of 0.78 a.u. However, it can be observed that the temperature with the slowest grain growth at 500 cycles of ZnO deposition was 200 °C, with a layer thickness of 60 nm. The device efficiency increased progressively with deposition temperature, reaching a maximum power conversion efficiency of 4.63% for ZnO films deposited at 300 °C with 500 ALD cycles. The observed enhancement is attributed to improved crystallinity, grain growth, and film uniformity at elevated deposition temperatures, which collectively enhance charge transport and reduce recombination losses. These results demonstrate that optimizing the ALD temperature is a key factor in achieving high-quality ZnO films and improved DSSC performance.

## 1. Introduction

In contrast to conventional silicon-based solar cells, dye-sensitized solar cells (DSSCs) are a more affordable and ecologically friendly photovoltaic technology. A DSSC typically comprises four key functional layers, each performing a distinct role in converting light into electricity. First, the transparent conductive substrate, typically glass-coated FTO or ITO, serves as the electrical contact and allows sunlight to enter the cell [1]. Second, a nanoporous wide-bandgap semiconductor photoanode which commonly uses Titanium Dioxide (TiO_2_), provides a high-surface-area scaffold for the dye, and acts as the medium for electron injection and transport [2].

Third, a photosensitizer dye layer adsorbed on the semiconductor absorbs incident photons, generates excited electrons, and injects them into the conduction band of the semiconductor [3]. Finally, a redox-active electrolyte (I^−^/I_3_^−^) and a counter electrode (usually a catalytic conductive layer such as Pt or carbon) complete the circuit by regenerating the dye and facilitating charge transfer, ensuring continuous operation under illumination. The photoanode’s semiconductor composition, which is essential to electron transport and overall device performance, is at the heart of DSSC efficiency [4]. Effective charge recombination suppression at semiconductor/electrolyte and semiconductor/dye interfaces is still a crucial strategy for enhancing long-term stability and device performance in DSSCs. Applying a thin passivation layer, which can reduce surface imperfections, favorably align energy levels, and act as a barrier to back-electron transfer, is one viable tactic [5,6]. Passivation coatings or treatments in photovoltaics eliminate interface and surface imperfections that operate as non-radiative recombination sites, hence enhancing charge-carrier lifespan and raising open-circuit voltage (V_o_c) and overall efficiency [7]. Recently, Tan et al. (2025) [8], by adding a thin AlN passivation layer onto Mg-doped β-Ga_2_O_3_, significantly reduced surface defect-related carrier recombination and interface trap losses, thereby enhancing photogenerated carrier collection. As a result, the metal–insulator–semiconductor–insulator–metal (MISIM) photodetector exhibits substantially improved responsivity, external quantum efficiency (EQE), and dark-current suppression relative to un-passivated devices [8].

According to reports, zinc oxide (ZnO) exhibits exceptional intrinsic electron mobility, with values between 200 and 300 cm^2^/Vs. This high electron mobility minimizes energy losses from electron recombination at defect sites by lowering resistance in the conduction channel, which greatly speeds up charge transfer. The energy conversion efficiency of DSSCs is directly improved by such effective charge-collecting techniques, which also result in increased photocurrent production [9]. ZnO’s distinct qualities make it appealing for use in DSSCs and photovoltaics in general. Although the band gap energies and electron affinities; 3.37 eV and 4.3 eV, respectively, of ZnO and TiO_2_ are similar, ZnO stands out because of its enormous exciton binding energy of 60 meV, high electron mobility measuring between a range of 200 and 300 cm^2^/Vs, and its significantly higher electron diffusivity values of 1.2 × 10^−2^ cm^2^/s [10,11]. Apart from being an appealing material for solar cell applications, ZnO is also reasonably priced and shows durability against photo-corrosion [12].

ZnO nanostructures are more suited for DSSCs due to their adaptability. In order to maximize electron transport and light harvesting, researchers have investigated a variety of morphologies, such as nanowires, nanorods, and hierarchical architectures. For example, straight electron-conduction paths provided by ZnO nanowires lower recombination losses and increase cell efficiency, with reported values ranging from approximately 1% to over 6% [13]. For light absorption and subsequent photoelectric conversion, these nanostructures’ large surface area also makes it easier to load more dye [14]. The formation of a uniform ZnO passivation layer over the primary semiconductor may enhance conduction-band alignment, decrease the surface trap state density, and increase electron lifespan. Higher open-circuit voltage (V_o_c), lower dark current, and enhanced fill factor values of 0.82 V, 0.43 mA/cm^2^, and 60.6%, respectively, are typically the result of these effects [15,16]. Emerging research on ZnO as a passivating or electron-selective interface layer in diverse solar systems shows its wider applicability, even if the majority of the work currently concentrates on ZnO as a main photoanode material [17]. For this study, the ZnO nanolayers are used as passivating layers that help prevent electrons from recombining with the electrolyte or oxidized dye molecules at the interface by acting as a blocking layer for the TiO2 semiconductor. Consequently, short-circuit current increases as a result of more electrons being gathered due to less recombination [5,18]. This scientific research investigated the influence of ALD parameters, specifically deposition temperature, to achieve optimal performance.

Even with these benefits, a major problem for ZnO-based DSSCs is ZnO’s chemical instability in acidic dye solutions since it might result in surface imperfections that trap electrons and speed up recombination [19]. To improve the stability and effectiveness of ZnO-based DSSCs, methods such as surface passivation, doping, and the creation of substitute dye molecules have been explored [20]. The precise and conformal thin-film deposition method known as atomic layer deposition (ALD), which makes it possible to create homogeneous thin-film layers at the atomic scale, was applied in this study. Compared to sputtering, sol–gel, or conventional chemical vapor deposition, which frequently produce thicker, non-conformal, or porous films prone to pinholes or uneven coverage, ALD-deposited ZnO layers maintain high interface quality and reproducibility, making them especially useful for photovoltaics and nanostructured devices [21,22]. Even on intricately nanostructured surfaces, ALD’s ability to regulate layer thickness and composition through consecutive, self-limiting surface reactions makes it possible. The influence of precursor and substrate temperature on the evolution of ZnO nanostructures during atomic layer deposition remains insufficiently understood, despite its central importance for controlling film quality and device performance. According to the literature, temperature governs precursor adsorption, surface reaction kinetics, ligand removal and ultimately, the crystallinity, stoichiometry, and defect density of the resulting ZnO layers. However, the optimal ALD temperature that allows slow grain growth, surface homogeneity, crystallinity, and the formation of a compact passivating ZnO nanolayer remains undetermined. This study aims to systematically determine how the deposition temperature affects the nucleation behavior, growth mechanism, and microstructural development of ZnO passivating nanolayers, with particular attention to grain size, orientation, porosity, and electrical properties. After the optimal deposition temperature is determined, by correlating these temperature-dependent structural changes with the photovoltaic response of the dye-sensitized solar cells (DSSCs), the work evaluates how ZnO microstructure influences charge transport, recombination dynamics, and overall device efficiency [23,24].

## 2. Materials and Methods

The preferred substrate was a Fluorine-doped Tin Oxide (FTO) glass slide L × W × thickness; 100 mm × 100 mm × 2.3 mm with surface resistivity of ~7 Ω/sq (Sigma Aldrich, Darmstadt, Germany), which functions as a transparent conducting oxide. These FTO glass substrates also have a transmission/permeability of 80–82% and thermal stability up to 640 °C. The cleaning agents were Ethanol and Isopropanol from Sigma Aldrich, Darmstadt, Germany. PTI platinum paste (Sigma Aldrich, Darmstadt, Germany) was applied onto the FTO glass substrate to serve as the counter electrode. The 18NR-AO Active Opaque Titania Paste (GreatCell Solar Materials, Queanbeyan, Australia) was deposited as the semiconductor layer to construct the working electrode. The EL-HSE High Stability Electrolyte (Sigma Aldrich, Darmstadt, Germany) was introduced to sensitize the active working electrode, while the N719 Industry Standard Dye (GreatCell Solar Materials, Queanbeyan, Australia) was employed as the sensitizing dye for the semiconductor material. These materials did not require any additional processing because they were all acquired at industrialized quality.

After being carefully taken out of their package, the 100 mm × 100 mm × 2.3 mm Fluorine Tin Oxide (FTO) glass substrates were then cut into samples that measured 2.5 mm × 2.5 mm square. The substrates were handled by their sides, taking care to avoid touching the region covered with FTO. The substrates are placed into sanitized glass beakers that have been filled with a small amount of detergent solution and deionized water. This will aid in the physical cleaning of the substrates. Following that, the substrates were thoroughly cleaned using deionized water. After that, the beaker was submerged in boiling deionized water in an ultrasonic bath and sonicated for fifteen minutes each. Rapid vibration around the substrates is produced by the ultrasonic bath, which helps to remove particles. After that, the substrates were washed and sonicated in isopropyl alcohol (IPA) and acetone. For 15 min, each procedure was subjected to further sonication. Prior to the Zinc oxide being deposited, the substrates were given time to dry.

The ALD Picosun R200 system (Espoo, Finland) was used to deposit ZnO thin films on the cleaned FTO substrates. Water (H_2_O) and Diethylzinc (DEZn) purchased from Strem Chemicals (Newburyport, MA, USA) were used as precursors. With different deposition temperatures of 100 °C, 200 °C, and 300 °C, pulse lengths of 0.1 s each for the formation of the DEZn and H_2_O precursors, standard thermal ALD parameters, and a pressure of 0.1 mbar (10 Pa) were employed. To remove extra precursors and reaction byproducts, a 4-s N_2_ purge was added in between pulses. During the precursor pulse, sufficient exposure ensures that all accessible surface-binding sites are occupied, preventing further reactions and thereby enforcing self-limiting adsorption. The purge step subsequently eliminates any excess precursors and volatile byproducts, eliminating gas-phase reactions that might disrupt the self-limiting characteristic. Chamber pressure (0.1 mbar) and flow characteristics (200 sccm) influence both efficient precursor delivery to the surface and effective purging, resulting in uniform surface saturation and clean residual gas removal. For each of the aforementioned deposition temperatures, 100, 200, 300, 400, and 500 cycles of deposition were employed. These temperatures were selected because, according to Cai et al. (2019) [25], the self-limiting process is hindered when DEZ + H_2_O is used at low substrate temperatures because of fewer reactive -OH sites, lesser mass gain, and inferior crystallinity. Under some circumstances, incomplete surface reactions at temperatures below around 100 °C might result in sub-stoichiometric ZnO or worse film quality [25].

A screen with a mesh size optimized for uniform film deposition was selected to ensure consistent layer thickness and pattern fidelity. The prepared screen was carefully aligned and positioned over the ZnO-coated FTO glass substrate. The titanium dioxide paste (18NR-AO Active Opaque Titania Paste, GreatCell Solar Materials, Queanbeyan, Australia) was then applied onto the surface of the screen. Using an automated squeegee system, the paste was uniformly distributed and pressed through the mesh openings, resulting in a controlled deposition of the TiO_2_ layer onto the ZnO-FTO substrate according to the predefined pattern. Following the screen-printing process, the deposited TiO_2_ film was subjected to a drying stage to remove residual solvents and promote adhesion between the TiO_2_ particles and the underlying ZnO-FTO substrate. This drying procedure was carried out in a temperature-controlled oven at 100 °C for 10 min, ensuring complete solvent evaporation and formation of a mechanically stable TiO_2_ layer suitable for subsequent device fabrication steps.

To enhance film adhesion, remove organic residues, and improve the overall electrochemical performance, the titanium dioxide layers deposited on ZnO-FTO glass substrates were subjected to a post-deposition thermal treatment. The samples were annealed in a heating oven at 480 °C for 30 min under ambient conditions. This heat treatment facilitates the removal of residual binders and organic components from the TiO_2_ paste, promotes interparticle necking and crystallization within the TiO_2_ network, and develops a mesoporous structure that enhances electron transport and dye adsorption capability.

Following the annealing process, the samples were allowed to cool gradually to room temperature to prevent thermal shock and preserve structural integrity. The resulting sintered TiO_2_ layer served as the photoanode in the device assembly. A parallel procedure was applied to separate FTO glass substrates coated with PTI Platinum paste (Sigma Aldrich, Darmstadt, Germany), which were similarly heat-treated under identical thermal conditions to achieve optimal conductivity and catalytic activity. These platinum-coated substrates functioned as the counter electrodes in the final device configuration.

The cleaned TiO_2_-coated ZnO-FTO glass substrates, serving as the working electrodes, were sensitized with dye by immersion in a 0.3 mM ethanolic solution containing the N719 ruthenium-based dye (GreatCell Solar Materials, Queanbeyan, Australia). The substrates were carefully placed in a sealed container filled with the dye solution and left to soak for 24 h under dark conditions to ensure uniform adsorption of dye molecules across the TiO_2_ surface. During this sensitization step, the carboxylate anchoring groups of the N719 dye formed coordination bonds with the surface titanium atoms of the TiO_2_ nanoparticles, enabling efficient electron transfer pathways for subsequent photoexcitation processes.

Upon completion of the adsorption period, the substrates were gently removed from the dye bath and rinsed with isopropanol to eliminate any non-chemisorbed dye molecules, thereby preventing dye aggregation and improving the reproducibility of the photoactive layer. The cleaned, dye-loaded electrodes were then allowed to dry under ambient conditions before cell assembly.

**Figure 1:**
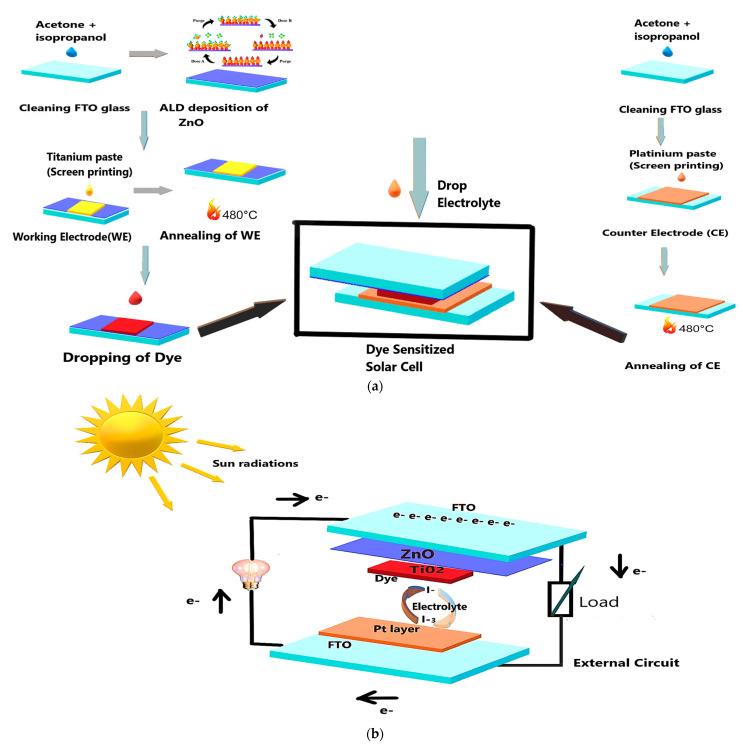
(**a**) Step-by-step fabrication of FTO-ZnO 500 dye-sensitized solar cells. (**b**) Components of FTO-ZnO dye-sensitized solar cells.

For device assembly, a small volume of EL-HSE High Stability Electrolyte (Sigma Aldrich, Darmstadt, Germany) was dispensed onto the active area of each dye-sensitized electrode using a precision syringe. This electrolyte served as the redox mediator, completing the internal circuit of the dye-sensitized solar cell (DSSC) and facilitating charge regeneration between the photoanode and counter electrode. A schematic representation of the fabrication procedure for the DSSCs is illustrated in Figure 1a and the completed device in Figure 1b.

## 3. Results and Discussions

### 3.1. Surface Morphology

The total performance of dye-sensitized solar cells (DSSCs) is significantly influenced by the surface shape and roughness of the materials utilized in these devices. These properties affect dye absorption, light adsorption, electron transport, and interfacial charge recombination, among other important elements of the cell’s operation. The efficient operation of DSSCs is greatly impacted by the shape of the photoanode material, which is usually made up of semiconductor nanoparticles like zinc oxide (ZnO). Increased dye molecule adsorption is made possible by a higher surface area provided by a highly porous and well-structured morphology. Increased light harvesting and increased photocurrent production are the results of this improved dye loading [1,26].

**Figure 2:**
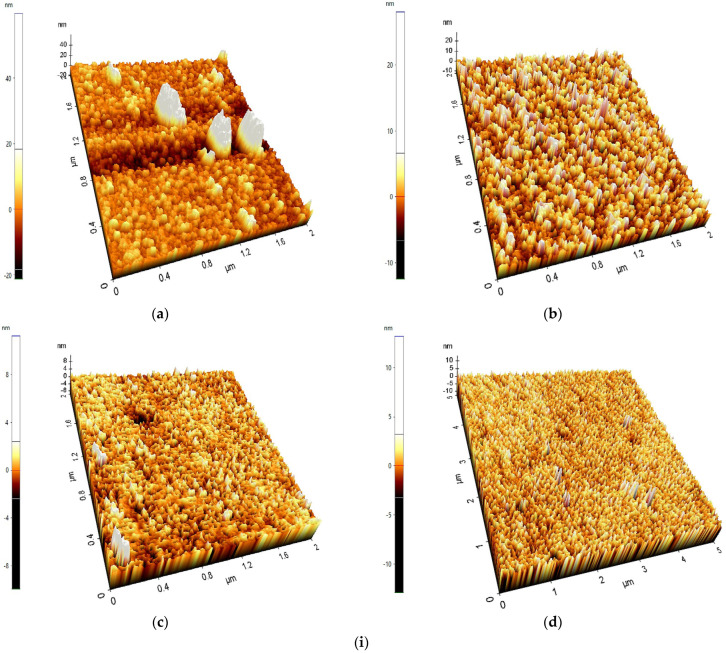

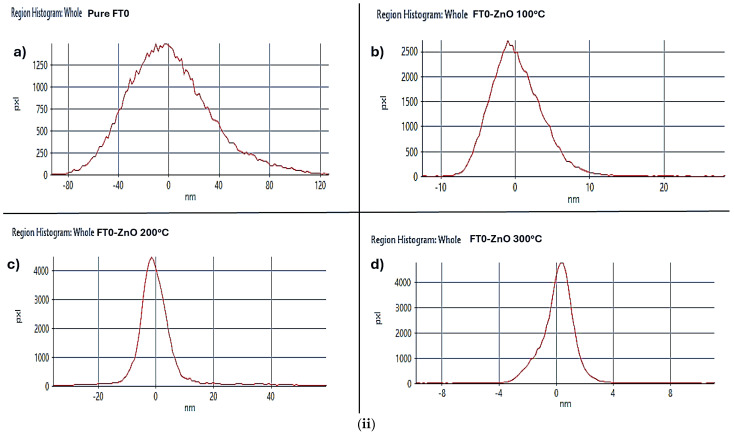
(**i**) 3D AFM topography of (**a**) Pure FTO glass, (**b**) FTO-ZnO 500 cycles-100 °C, (**c**) FTO-ZnO 500 cycles-200 °C, and (**d**) FTO-ZnO 500 cycles-300 °C. (**ii**) Particle Distribution Histogram: (**a**) Pure FTO glass; (**b**) FTO-ZnO 500 cycles-100 °C; (**c**) FTO-ZnO 500 cycles-200 °C; (**d**) FTO-ZnO 500 cycles-300 °C.

Figure 2i(b–d) is a surface morphology image obtained from the FTO-ZnO samples with the thickest layer (500 cycles), while Figure 2i(a) is the surface morphology image of a pure FTO glass using Atomic Force Microscopy. From the literature, the more crystalline but well-structured a semiconducting thin film is, the greater the dye-sensitized solar cell’s efficiency and light-harvesting capacity. Figure 2i(b) shows a surface with significantly large rod-like spikes; however, the surface is not crystalline or well-structured compared to the other morphologies obtained [27]. This could be due to a difference in deposition temperature. The trend in these diagrams is that surface morphology and structure increase significantly with increasing temperature, because temperature is critical for activating gas-phase reactions [28]. Figure 2i(d) shows a defined surface structure with fairly even crystalline structures that can easily absorb dye due to the available surface area. Higher temperatures often result in smoother, more crystalline deposits and faster deposition rates. It can be concluded that temperature has a significant effect on ZnO metal oxide deposition in ALD, influencing growth rate, crystallinity, and film characteristics. In general, higher temperatures and slower film development are encouraged, and ZnO metal oxide can be uniformly deposited onto a substrate by increasing the temperature to 300 °C.

Figure 2ii provides a particle distribution histogram of Pure FTO glass, FTO-ZnO 500 cycles-100 °C, FTO-ZnO 500 cycles-200 °C, and FTO-ZnO 500 cycles-300 °C. In Figure 2ii(a), the FTO substrate shows large topographical variations (−80 nm to +120 nm). This is typical because commercial FTO is polycrystalline with large grains and step edges. A broad height distribution indicates a rough native texture. At FTO-ZnO 500 cycles-100 °C, the ZnO layer partially smooths the substrate. The height distribution becomes more concentrated around 0 nm. This suggests partial filling of valleys and coverage of FTO grain features, but not full smoothening. The ZnO film has become significantly smoother. At FTO-ZnO 500 cycles, 200 °C, the narrow height distribution suggests better film uniformity and more complete coverage of the underlying FTO. Obtaining smoother ZnO ensures fewer recombination sites at surface defects. At FTO-ZnO, 500 cycles, 300 (−6 to +6 nm), the surface is now highly smooth, with a histogram sharply peaked and very narrow. This indicates significant surface smoothing and a highly uniform film. This corresponds to more complete crystallization and grain coalescence at higher temperature [29]. Over a given sampling length, Ra is the average of the surface profile’s absolute departures from the mean line. The square root of the mean of the squared deviations of the surface profile heights from the mean line is Rq, sometimes referred to as RMS roughness. The lower the values of both Rq and Ra, the smoother the thin film surface [30]. In Table 1, it can be observed that FTO-ZnO 500 cycles-300 °C samples recorded the lowest Rq and Ra values of 1210 nm and 0.877 nm, respectively. These values show that when the ALD temperature increases, the growth rate decreases, leading to more homogeneous deposition and a more crystalline, smoother surface. This result is optimal; however, if the deposition temperature is too high (for example, above 350 °C), the surface would be too smooth, decreasing light and dye absorption, which can then negatively affect the performance of the dye-sensitized solar cells [31].

The surface morphology and microstructure of dye-sensitized solar cells (DSSCs) was studied with the use of Carl Zeiss Supra 35 scanning electron microscope (SEM) purchased from (Oberkochen, Germany). SEM can create finely detailed images of the electrodes used in DSSCs, such as the photoanode and counter electrode.

**Figure 3:**
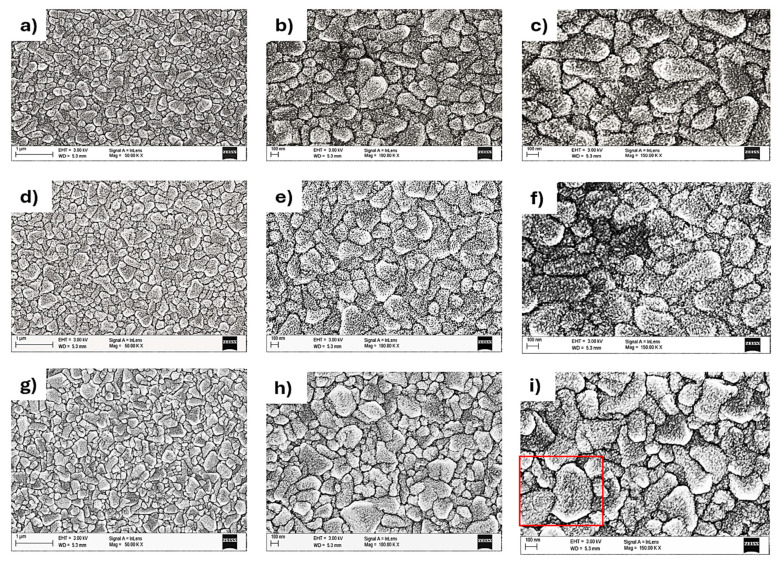
SEM images of (**a**) FTO-ZnO 500 cycles-100 °C (50.00 KX) (**b**) FTO-ZnO 500 cycles-100 °C (100.00 KX) (**c**) FTO-ZnO 500 cycles-100 °C (150.00 KX) (**d**) FTO-ZnO 500 cycles-200 °C (50.00 KX) (**e**) FTO-ZnO 500 cycles-200 °C (100.00 KX) (**f**) FTO-ZnO 500 cycles-200 °C (150.00 KX) (**g**) FTO-ZnO 500 cycles-300 °C (50.00 KX) (**h**) FTO-ZnO 500 cycles-300 °C (100.00 KX) (**i**) FTO-ZnO 500 cycles-300 °C (150.00 KX).

Figure 3 clearly shows that FTO-ZnO 500 cycles-300 °C samples exhibit a more homogenous and crystalline surface microstructure as compared to FTO-ZnO 500 cycles-100 °C and FTO-ZnO 500 cycles-200 °C samples. Temperature significantly affects ZnO nanolayers by influencing their morphology, crystallinity, and even optical properties. Higher temperatures generally lead to improved crystallinity and changes in particle shape, while also causing a decrease in the band gap and affecting defect states [32] FTO-ZnO 500 cycles-300 °C (150.00 KX) shows a more crystalline, well-structured surface morphology compared to FTO-ZnO 500 cycles-200 °C and FTO-ZnO 500 cycles-100 °C because temperature affects deposition of ZnO in ALD, hence ZnO growth requires a delicate balance between enough thermal energy to make each half-reaction complete, and low enough temperature that DEZn and the oxidant do not decompose or desorb too quickly [33]. It can be deduced that the deposition temperature of 300 °C falls within the temperature range where there is a balance between the thermal energy for the half-reactions and precursor absorption, resulting in more uniform and conformal grain growth compared to those deposited at much lower temperatures. FTO-ZnO 500 cycles-200 °C and FTO-ZnO 500 cycles-100 °C exhibit less uniform or crystalline surface morphology because if the substrate is not hot enough, the first precursor (DEZn) may adsorb incompletely or reversibly, leaving fewer Zn sites for the next oxidant step. This reduces growth per cycle, leading to less crystalline or structural morphology [34]. Again, as discussed and shown in the Surface Roughness parameters and AFM images above, SEM microstructure results show that the higher the deposition temperature, the more defined the crystal structure. This shows that the deposition temperature during ALD is crucial for forming a uniform, crystalline, and smooth thin film of ZnO semiconductor layers in the fabrication process of a DSSC anode [20,35].

Using energy-dispersive X-ray spectroscopy (EDS), the elemental makeup and spatial distribution of important elements in the samples were examined. An SEM with an EDS detector was used for the investigation, enabling the simultaneous collection of compositional and morphological data. The distinctive X-rays released when the sample is exposed to an electron beam are detected using EDS mapping, which yields semi-quantitative information on the existence and relative abundance of elements [36]. For the purpose of evaluating the material’s homogeneity and visualizing the distribution of each pertinent species over the sample surface, elemental maps were created. Phase creation, compositional segregation, and interactions between various layers or components were identified through the co-localization of elements in overlay maps. Semi-quantitative elemental concentration comparisons were supported by spot and area studies [37].

Thus, EDS analysis in this work fulfilled two functions: it verified that ALD successfully deposited ZnO and showed the spatial correlation of elements, providing information on the sample’s microstructure pertinent to its functional performance.

**Figure 4:**
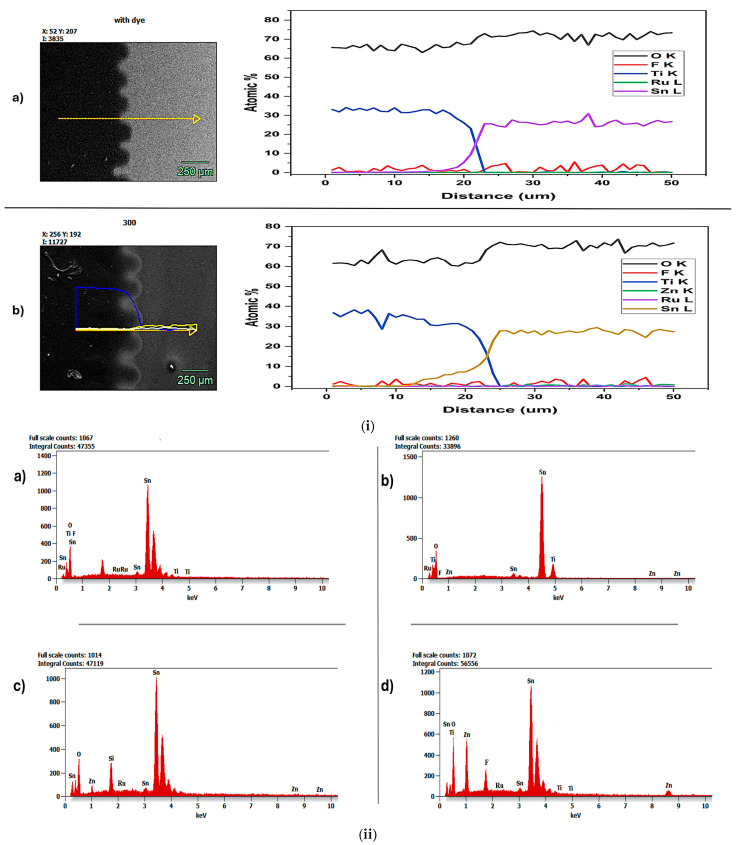
(**i**) EDS mapping analysis images of (**a**) pure FTO photoanode with dye and (**b**) ZnO-FTO 500 cycles-300 °C photoanode with dye. (**ii**) EDS Elemental count analysis of (**a**) pure FTO photoanode with dye, (**b**) ZnO-FTO 500 cycles-100 °C photoanode with dye, (**c**) ZnO-FTO 500 cycles-200 °C photoanode with dye, and (**d**) ZnO-FTO 500 cycles-300 °C photoanode with dye.

As shown in Figure 4i(a), the obtained mapping image clearly distinguished the TiO_2_ and FTO layers. As shown in the image, black (TiO_2_) and gray (FTO) distinct layers exhibit non-homogeneity, as observed in the AFM analysis shown in Figure 2i(a). The elemental count graph shown in Figure 4i(a) shows Oxygen (red), Fluorine (green), Titanium (blue), Ruthenium (yellow), and Tin (white), indicating that the samples contained only FTO, TiO_2_, and a ruthenium-based N719 dye. Hence, no ALD-deposited ZnO or metal oxide was observed, resulting in the absence of a well-structured morphology, as confirmed by AFM analysis.

However, in Figure 4i(b), the ZnO-FTO 500 cycles-300 °C photoanode with dye obtained a mapping image that shows a distinction between the layers; however, there is not a clear difference between the semiconductor layer and the ZnO deposited via ALD. This is because at 300 °C, the temperature has a significant effect on ZnO metal oxide deposition in ALD; it affects growth rate, crystallinity, and film characteristics, thereby causing a homogeneous morphology. The elemental count graph shown in Figure 4i(b) is Oxygen (red), Fluorine (green), Titanium (blue), Zinc (yellow), Ruthenium (white), and Tin (wine). This indicates that ZnO was successfully deposited via ALD due to the presence of Zn. Again, the elemental count graph in Figure 4i(b) shows greater overlap of the plots, indicating interactions between various layers or components. In contrast, the elemental count graph in Figure 4i(a) shows less overlap, indicating greater compositional segregation [38].

Figure 4ii shows the elemental count peaks in (a) pure FTO photoanode with dye, (b) ZnO-FTO 500 cycles-100 °C photoanode with dye, (c) ZnO-FTO 500 cycles-200 °C photoanode with dye, and (d) ZnO-FTO 500 cycles-300 °C photoanode with dye. Though all ALD temperatures record the presence of Zn, ZnO-FTO 500 cycles-300 °C exhibits the highest continuous peak of Zn as compared to those deposited at 100 °C and 200 °C. This is possibly due to the slow grain growth at 300 °C, which ensures uniform, compact ZnO nanolayers. The more uniform and compact the passivating layer, the less likely it is to be dissolved by the Ruthenium-based N719 acidic dye (Ru), which usually has a pH of 3.8 [39]. These findings imply that the morphological structure helps maintain functional integrity, as also suggested by Kumara et al. (2018) [40]. Again, using a low concentration (0.3 mM) of the ethanolic N719 dye bath might also have contributed to the relative stability of the ZnO nanolayer, even in the presence of an acidic bath, as further explained in Giannouli et al., 2018 [9]. However, Figure 4ii(a) does not record the presence of Zn because this is the pure FTO sample without an ALD-deposited ZnO passivating nanolayer.

### 3.2. Light Transmittance Analysis

The efficiency and quality of different optical materials and systems are directly impacted by transmittance. Since the anode of a Dye-Sensitized Solar Cell needs to be transparent or translucent to absorb sunlight, the desired characteristics in terms of light transmittance and absorption are high [41,42].

**Figure 5:**
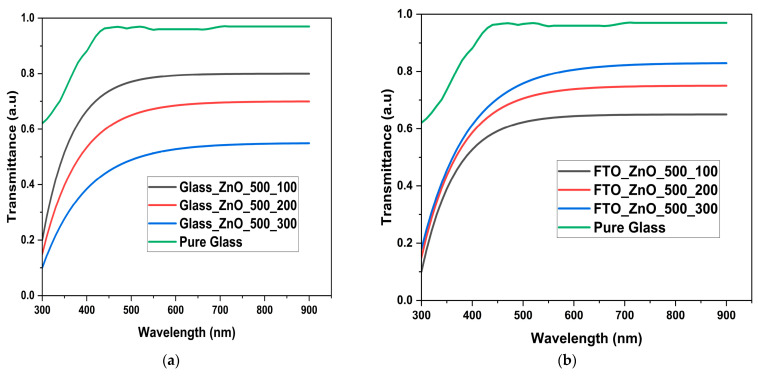
(**a**) Transmittance graph of Pure glass, glass-ZnO-500 cycles-100 °C, glass-ZnO-500 cycles-200 °C, glass-ZnO-500 cycles-300 °C (**b**) Pure glass, FTO-ZnO-500 cycles-100 °C, FTO-ZnO-500 cycles-200 °C, FTO-ZnO-500 cycles-300 °C.

Figure 5 displays transmittance plotted against wavelength for glass, ZnO deposited on glass with varying temperatures, and ZnO deposited on FTO with varying temperatures. The graph (on the right) shows that pure glass samples have the highest transmittance within 250–950 nm, followed by FTO-ZnO-500 cycles-300 °C, then FTO-ZnO-500 cycles-200 °C, and finally FTO-ZnO-500 cycles-100 °C. Generally, all samples with ZnO nanostructures exhibit a trajectory similar to that of pure glass at 500 nm. Therefore, they are all fairly transparent, absorb light over a wide wavelength range, and allow light to pass through and disperse. However, the FTO-ZnO-500 cycles-300 °C samples follow closely behind because the refractive index is determined by the surface roughness parameters (Table 1) and by surface homogeneity. This is because temperature slows growth, producing smooth, crystalline surfaces that facilitate easier light absorption.

FTO-ZnO-500 cycles-200 °C samples exhibited lower transmittance because, as seen in the particle distribution graphs and AFM, they were less homogeneous, although not as significant as the samples at 300 °C [3]. However, a much more distinct and opposite trend was observed with only glass-ZnO samples. This could be due to the absence of conductive FTO layers that react with ZnO to form oxygen bridges (Sn–O–Zn) or interfacial oxide linkages. These are polar covalent bonds between Zn and O atoms at the interface, forming a continuous oxide network [41]. This formed oxide interface reduces the transmittance of the FTO-ZnO-500 cycles samples, but, as can be seen in the results, an increase in heat treatment (deposition temperature) helps improve its transmittance or absorption.

### 3.3. Reflectometry and Thickness Analysis

Reflectometry was used to describe the deposited thin films’ optical characteristics and thickness. The strength of reflected light is measured as a function of wavelength or angle using this approach, which involves directing a collimated laser beam onto the sample surface. Light reflected from the film surface and the film–substrate contact interacts, causing variations in reflectance. It is possible to calculate the film thickness and refractive index with great precision by examining these interference patterns. Through the quantitative analysis of layer uniformity, reflectometry makes it possible to detect gradients or differences in thickness throughout the substrate. Correlating device performance with film morphology and deposition conditions requires this knowledge. To further enhance the accuracy of later functional tests like photovoltaic efficiency or optical absorption, reflectometry may be used to confirm the completeness of each deposition stage and to guarantee repeatability between samples [42].

**Figure 6:**
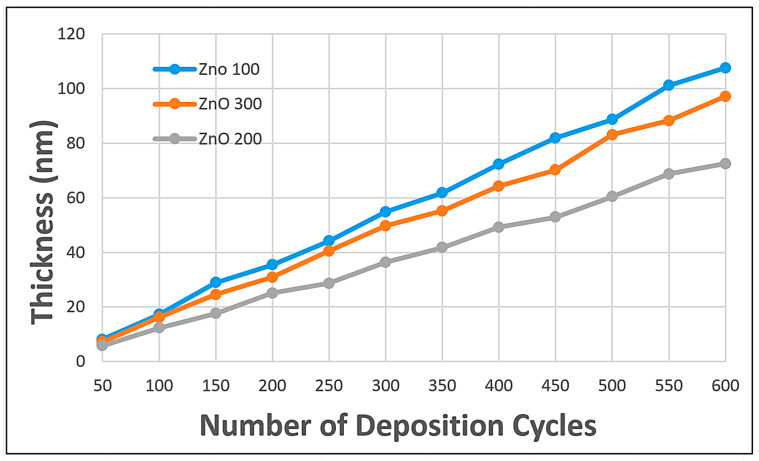
Reflectometry test on ZnO with varying temperatures and deposition cycles.

Figure 6 shows the growth trend of ZnO semiconducting nanostructures with varying deposition temperatures. This result aligns perfectly with the transmittance values recorded above, suggesting that the layer thickness is heavily affected by the deposition temperature. It is observed that, despite a similar number of deposition cycles, ZnO samples deposited at 100 °C have the thickest layer, confirming that the ZnO growth rate is overly rapid, thereby creating a bulky, less homogeneous, low-transmittance thin film [20,28].

However, a massive change is observed: ZnO deposited at 300 °C forms a thicker layer than that deposited at 200 °C, despite the higher temperature. This can be accounted for by the logic that, though increased temperature is beneficial in some respects, the appropriate number of deposition cycles should be considered [31]. According to Cai et al. (2019), increasing the deposition temperature during Atomic Layer Deposition (ALD) of ZnO does not always result in a straightforward increase in film thickness per cycle, as multiple competing factors influence growth. While moderate temperatures within the ALD window (~125–175 °C) favor self-limiting reactions with consistent growth per cycle, higher temperatures can induce desorption of intermediate precursor species and dehydroxylation of surface (−OH) groups, reducing the number of reactive sites [25]. Simultaneously, Mishra et al. (2021) reported that elevated temperatures promote grain growth, densification, and microstructural reorganization, which can alter film packing and result in an apparently thicker layer despite fewer ideal monolayer depositions [43]. Parasitic reactions or partial CVD-like behavior may also contribute at higher temperatures, further complicating thickness evolution [44]. Therefore, optimizing both deposition temperature and the number of ALD cycles is critical to achieve controlled, high-quality ZnO passivation nanolayers with desirable structural and functional properties. From Figure 6, it can be observed that at 500 cycles of deposition, ZnO-200 °C has a layer thickness of 60 nm, whereas ZnO-300 °C has a layer thickness of 83 nm. Assuming 60 nm is the optimal layer thickness for a DSSC, the graph shows that ZnO-300 °C must undergo 400 deposition cycles.

### 3.4. Electrical Performance and Solar Simulation Analysis

A solar simulator that mimics typical sunshine circumstances was used to assess the developed dye-sensitized solar cells’ (DSSCs’) photovoltaic performance. As advised by the International Electrotechnical Commission (IEC 60904-3) for terrestrial photovoltaic testing, the system used a Solar Cell I–V Tracer System (PV Test Solutions Tadeusz Zdanowicz, Wrocław, Poland) and a Keithley 2400 source meter (Tektronix, Beaverton, OR, USA) under standard AM 1.5 radiation and a light intensity of 1000 W/m^2^. In order to provide precise and repeatable measurements, the simulator produced a steady and consistent light throughout the active cell region. Current–voltage (J–V) characteristics were collected under simulated sunshine to estimate important performance metrics, such as overall power conversion efficiency (PCE), fill factor (FF), open-circuit voltage (V_o_c), and short-circuit current density (Jsc). The processes of charge creation, transport, and collection inside the cell are quantitatively revealed by these characteristics. The evaluation of cell stability and repeatability is also made possible by solar simulation, which compares device performance under regulated and constant light circumstances. By enabling meaningful comparisons between various production settings and material compositions, the use of a calibrated solar simulator thereby offers a dependable and consistent way for assessing the efficiency and functional behavior of DSSCs [26].

**Figure 7:**
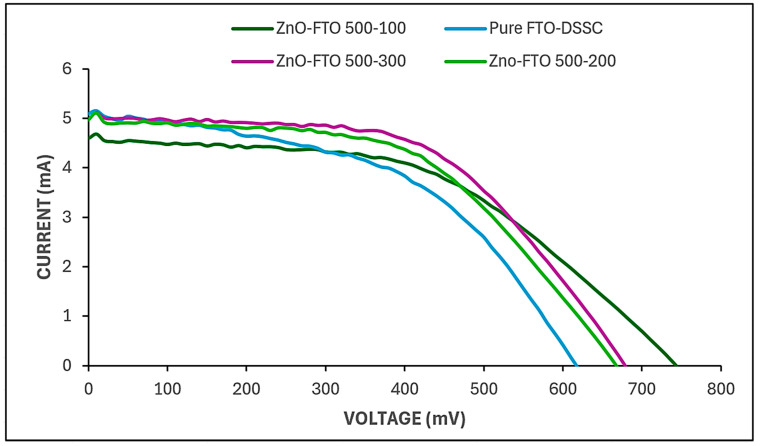
Solar simulation of pure FTO, FTO-ZnO 500 cycles-100 °C, FTO-ZnO 500 cycles-200 °C, and FTO-ZnO 500 cycles-300 °C dye-sensitized solar cells.

Figure 7 and Table 2 illustrate the clear positive influence of atomic layer deposition (ALD) temperature on the photovoltaic performance of dye-sensitized solar cells (DSSCs). The reference device, consisting of a bare fluorine-doped tin oxide (FTO) substrate without any additional metal oxide layer, exhibited a power conversion efficiency (PCE) of 3.76%, which is consistent with the typical efficiency range reported for FTO-based DSSCs [8]. Upon the introduction of ALD-deposited ZnO nanolayers onto the FTO substrate, a notable enhancement in device performance was observed. Specifically, the DSSC fabricated with ZnO deposited at 100 °C showed a 0.45% absolute increase in efficiency compared to the pristine FTO cell. This improvement demonstrated a distinct trend: as the ALD temperature increased, the device efficiency correspondingly improved. The sample fabricated with 500 ALD cycles of ZnO at 300 °C achieved the highest PCE of 4.63%. This indicates that, even under identical deposition cycles, the deposition temperature exerts a significant effect on the structural and functional quality of the ZnO films, thereby influencing the overall photovoltaic response of the DSSC [42].

The observed enhancement in efficiency with increasing deposition temperature can be attributed to the influence of temperature on the ZnO film’s microstructural evolution, specifically, grain growth dynamics, crystallinity, and film uniformity. Elevated temperatures promote improved crystallinity and denser film formation due to enhanced atomic mobility during the ALD process. These conditions favor the development of larger, well-oriented ZnO grains with fewer structural defects, which in turn facilitate more efficient charge transport and reduced recombination losses at the semiconductor–electrolyte interface [45]. Furthermore, when the two oxides (ZnO and TiO_2_) form an interface, their Fermi levels align through interfacial charge transfer or band bending, setting a common chemical potential; this forces band bend such that electrons accumulate in the lower-energy conduction band (TiO_2_) under illumination, which promotes charge separation and suppresses back-transfer hence contributing to the total cell efficiency [46]. Correlating the results from the particle size distribution, AFM, reflectometry, and electrical performance, it can be deduced that the thickness of a ZnO layer in a DSSC is a critical parameter that influences multiple aspects of device performance. On one hand, increasing the deposition temperature (300 °C) fairly increases the ZnO layer thickness and crystallinity, which consequently increases the available compact blocking layer that prevents electrons from recombining with electrolyte or oxidized dye molecules at the interface. A thicker layer thus often leads to higher light absorption (due to greater dye loading) and potentially enhanced short-circuit current density (I_s_c) of 5.039 mA, because more photons can be harvested and more electrons injected into the semiconductor conduction band [47]. However, if the blocking layer is too thick, for example, in the case of FTO-ZnO 500 cycles-100 °C, the distance electrons must travel from their injection sites (dye–ZnO-TiO_2_ interface) to the conductive substrate (FTO) increases, which lengthens the electron diffusion path, reducing overall power conversion efficiency (η) [48]. The V_o_c for FTO-ZnO 500 cycles-300 °C was lower (678.429 mV) compared to the V_o_c at 100 °C (743.181 mV). This is because improved crystallinity (for example, single-crystal nanowires or well-sintered polycrystalline films) improves electron mobility and diffusion length because there are fewer scattering sites (grain boundaries, defects) delaying or trapping electrons. However, occasionally “improving crystallinity” (e.g., by high-temperature annealing) lowers surface area or dye adsorption capacity, which reduces short-circuit current (I_s_c), potentially harming overall efficiency [49].

In summary, higher ALD temperatures, particularly around 300 °C, result in the formation of more uniform and crystalline ZnO layers on the FTO substrate. This structural optimization directly contributes to the improved photoelectronic properties and enhanced power conversion efficiency of the corresponding DSSCs.

## 4. Conclusions

This study shows that ALD-grown ZnO layers significantly enhance DSSC performance when the deposition temperature is optimized. Increasing the temperature from 100 °C to 300 °C produced progressively smoother and more crystalline ZnO films, confirmed by AFM roughness values decreasing from Rq = 9.089 nm and Ra = 5.143 nm (100 °C) to Rq = 1.210 nm and Ra = 0.877 nm (300 °C). SEM imaging further verified that 300 °C yields the most uniform grain structure, while EDS mapping confirmed the strongest continuous Zn signal at this temperature, indicating well-formed, compact layers. Optically, higher-temperature films exhibited improved transparency and more controlled thickness. Reflectometry showed that although all samples used 500 cycles, ZnO thickness varied strongly with temperature: ~60 nm at 200 °C versus ~83 nm at 300 °C, demonstrating the strong temperature dependence of growth rate and density. These structural refinements directly improved photovoltaic behavior. The unmodified FTO cell produced an efficiency of 3.76%, while ZnO-FTO devices increased to 4.21% (100 °C), 4.34% (200 °C), and finally 4.63% at 300 °C. The 300 °C sample also achieved the highest FF (0.55), Pmax (1.888 mW), and maintained a high Jsc (5.039 mA), showing reduced recombination and more efficient charge extraction.

This study demonstrates an improvement of 0.87% (from 3.76% to 4.63%) using the same number of ALD cycles, emphasizing that deposition temperature alone can strongly tune microstructure and device performance. The difficulty of obtaining high performance with ZnO alone is highlighted by the fact that many contemporary ZnO-based DSSCs barely surpass 1–3% PCE [50]. The positive improvements are usually less than 1%; this adds relative weight to the findings in this study, attaining 4.6% PCE with ZnO via ALD.

The results confirm that 300 °C delivers the optimal balance between smoothness, compactness, crystallinity, and optical quality, establishing clear processing parameters for high-quality ZnO blocking layers in DSSCs. Although the ethanolic dye bath concentration and surface morphology helped ZnO deposition at 300 °C, it is suggested that further doping the passivating nanolayers with elements stable in acidic conditions, such as Titanium, would drastically improve the chemical stability of the ZnO layers, thereby potentially increasing the electrical performance of the DSSC.

## Figures and Tables

**Table 1:** Roughness Parameters measured for FTO-ZnO 500 cycles-100 °C, FTO-ZnO 500 cycles-200 °C, and FTO-ZnO 500 cycles-300 °C.

Region (Whole) [µm]	Max [nm]	Min [nm]	Rq [nm]	Ra [nm]
Pure FTO (2 × 2)	126.514	−92.932	33.001	25.951
FTO-ZnO 500 cycles-100 °C (2 × 2)	57.849	−18.955	9089	5143
FTO-ZnO 500 cycles-200 °C (2 × 2)	29.002	−10.103	3419	2698
FTO-ZnO 500 cycles-300 °C (2 × 2)	11.195	−9920	1210	0.877

**Table 2:** Measured solar simulation parameters and efficiencies.

Sample	Isc (mA)	V_o_c (mV)	Imax (mA)	Vmax (mV)	Pmax (mW)	FF (-)	Efficiency (%)
Pure FTO DSSC	5.084	616.898	3.737	409.261	1.529	0.49	3.76
FTO-ZnO 500 cycles-100 °C	4.602	743.181	3.737	456.731	1.707	0.50	4.21
FTO-ZnO 500 cycles-200 °C	4.959	667.453	4.157	425.694	1.769	0.53	4.34
FTO-ZnO 500 cycles-300 °C	5.039	678.429	4.273	441.878	1.888	0.55	4.63

## Data Availability

Data are contained within this article.

## Abbreviations

The following abbreviations are used in this manuscript:

ALD	Atomic layer deposition
DSSCs	Dye-sensitized solar cells
DEZn	Diethylzinc
FTO	Fluorine Tin Oxide
IPA	Isopropyl alcohol
Pt	Platinum
TCOs	Transparent Conducting Oxides
TiO_2_	Titanium dioxide
ZnO	Zinc Oxide
